# How to deal with low-flow low-gradient aortic stenosis and reduced left ventricle ejection fraction: from literature review to tips for clinical practice

**DOI:** 10.1007/s10741-021-10090-0

**Published:** 2021-03-08

**Authors:** F. Contorni, M. Fineschi, A. Iadanza, A. Santoro, G. E. Mandoli, M. Cameli

**Affiliations:** Department of Medical Biotechnologies, Division of Cardiology, University of Siena, Siena, Italy

**Keywords:** Low-flow low-gradient aortic stenosis, TAVI in low-flow low-gradient aortic stenosis, TAVI in bicuspid aortic valve, After TAVI paravalvular leak, After TAVI permanent pacemaker implantation, SAVR vs TAVI

## Abstract

Low-flow low-gradient aortic stenosis (LFLG AS) with reduced left ventricle ejection fraction (LVEF) is still a diagnostic and therapeutic challenge. The aim of this paper is to review the latest evidences about the assessment of the valvular disease, usually difficult because of the low-flow status, and the therapeutic options. Special emphasis is given to the available diagnostic tools for the characterization of LFLG AS without functional reserve at stress echocardiography and to the factors that clinicians should evaluate to choose between surgical aortic valve repair, transcatheter aortic valve implantation, or medical therapy.

## Introduction

Low-flow low-gradient aortic stenosis (LFLG AS) is an echocardiographic entity defined by a mismatch between a reduced aortic valve area (AVA, < 1 cm^2^) and a non-severe increase of transvalvular mean pressure gradient (MPG, < 40 mmHg) with an impaired stroke volume at rest (SV index ≤ 35 ml/m^2^) [1, 2]. LFLG AS still puts the clinicians in front of diagnostic and therapeutic dilemmas: making a correct diagnosis and choosing between an aortic valve replacement (AVR) on top of medical therapy vs an optimal medical therapy (OMT) alone. The choice passes through the balance between a key selection of those patients who can really benefit from AVR and the intrinsic risk related to the procedure. Finally, when AVR is the chosen treatment, surgical AVR (SAVR) and transcatheter aortic valve implantation (TAVI) are both available options. The aim of this paper is to review the latest evidences about the optimal diagnostic workup and management of LFLG AS with reduced left ventricle ejection fraction (LVEF < 50%).

## Severity assessment

Identifying the patients who might benefit from a valvular prosthesis rather than from OMT alone is a crucial step in the management of left heart valvular diseases. The main point is the identification of the right timing for intervention: not too early, to avoid the unnecessary exposition to procedural risks, but not too late, when heart could already be irreversibly damaged. According to the latest guidelines [1, 2], the assessment of a severe AS is the *sine qua non* to take AVR into consideration. While a suspected AS in the context of a normal flow status (classically defined by a normal stroke volume) can be defined as severe by echocardiography at rest (AVA < 1 cm^2^ and MPG > 40 mmHg), LFLG AS needs further diagnostic exams.

### Echocardiography

The combination of a LVEF < 50%, an AVA < 1 cm^2^, and an MPG < 40 mmHg can be explained by an underlying impaired left ventricle, unable to generate a sufficient SV for a complete AV opening, and potentially, a severe transvalvular gradient. Rest hemodynamic conditions do not provide a reliable evaluation of true AV performance when EF is reduced, so the actual guidelines [1, 2] recommend to perform a low-dose dobutamine stress echocardiography exam (DSE) to induce an SV increase and contemporarily analyze the AV adaptation. DSE can give three possible results:

- Significant increase in SV (∆SV ≥ 20%) and persistent low MPG (MPG < 40 mmHg): *pseudo-severe AS.* In this case, the LV contractile dysfunction outweighs AS inside heart failure (HF) pathophysiology. AS does not meet the hemodynamic criteria to be defined as severe.
- Significant increase in SV (∆SV ≥ 20%) and in MPG (MPG > 40 mmHg): *true severe LFLG AS*. Aortic valve is severely stenotic and the low gradient measured at rest is a consequence of the LV contractile dysfunction.
- Absence of significant increase in SV (∆SV < 20%) and MPG (MPG < 40 mmHg): *LFLG AS without functional reserve (FR).* In this case, DSE fails to demonstrate an AV hemodynamic adaptation to higher SV and the AS severity grade remains undetermined.

According to the current guidelines [1, 2], if DSE does not demonstrate a FR, the actual degree of stenosis cannot be assessed by dynamic echocardiographic parameters and clinicians have to rely on the morphologic features of the AV showed by cardiac computed tomography (CCT). In the following section, we describe the emerging role of two flow rate-based echocardiographic tools, although not already included in the diagnostic workup recommended by ESC and AHA.

### Echocardiography beyond functional reserve: the emerging role of the flow rate over the stroke volume

In recent years, new echocardiographic tools have been studied to overcome the diagnostic limitation of the ultrasound in the context of LFLG AS. In 2006, a large multicenter observational study on LFLG AS developed and validated a new echocardiographic tool to obtain reliable information about the severity of AS, even without FR at DSE: the projected aortic valve area (AVAProj) [3]. This parameter is intended to predict the value that AVA would reach at a normalized flow rate of 250 ml/s, basing on the AVA adaptations recorded during DSE at different flow ratio. AVAProj quantification assumes that, despite that DSE does not show a significant FR, the dobutamine infusion determines an increase of SV, although small. The assessment of AVA by the continuity equation at different flow ratios allows to estimate the so-called “valve compliance” (VC) and, as a result, the AVA in case of a normal SV. The original equation for AVAproj was

$$\mathrm{AVAproj }=\mathrm{ AVArest }+\mathrm{VC }\times (250 -\mathrm{ Qrest})$$

where AVArest is aortic valve area at rest and Qrest is transaortic flow rate measured at rest.

Recently, a simpler and less time-consuming equation has been validated [4]:

$$\mathrm{AVAproj}=\mathrm{AVArest}+(\triangle\mathrm{AVA}/\triangle\mathrm Q)\times(250-\mathrm{Qrest})$$

where ΔAVA is the difference between aortic valve area calculated by continuity equation at peak DSE and rest and ΔQ the difference between transaortic flow rate measured at peak DSE and rest.

In the latter formula, only basal left ventricle outflow tract (LVOT) diameter, LVOT velocity-time integral (VTI), AVA VTI at baseline and at DSE peak are needed to quantify the AVAproj. The reliability of this parameter has been recently confirmed by an update of the True or Pseudo Severe Aortic Stenosis (TOPAS) study [5] where AVAProj had a better diagnostic accuracy than MPG and AVA at peak DSE in detecting a severe AS: an AVAproj ≤ 1 cm^2^ showed a diagnostic accuracy (percentage of correct classification) of 70% in predicting severity while an aortic MPG ≥ 40 mmHg alone or combined with peak AVA ≤ 1 cmq of 48% and 47% only, respectively.

Thus, AVAproj is a promising tool to optimize the achievable data with DSE and brought to analyze the diagnostic predicting value of transaortic flow rate (FLR) at rest. Transaortic flow rate is defined as

$$\mathrm{FLR }=\mathrm{ SV}/\mathrm{LVET}$$

where LVET is the left ventricle ejection time, measured as the time interval between the beginning point and the end point of the LVOT VTI. Conceptually, FLR is a measure of flow (volume unit per unit of time) as well as stroke volume; nevertheless, the stroke volume measures the transaortic flow during a single cardiac beat (including both systole and diastole), while FLR measures only the effective blood volume ejected during the systolic phase. In a setting of low flow (SVi < 35 ml), FLR can be normal or reduced as a consequence to a short or long ejection time, respectively. A pioneering study showed that in a setting of low SV, a normal FLR at rest could be a good predictor of true severe AS because AVA will likely not increase during DSE; at the contrary, when the rest FLR is < 200 ml, DSE can be truly useful to unmask a pseudo-severe LFLG AS [6].

### Cardiac computed tomography

There is an extensive body of research showing CCT as a reliable test to detect severe AS. CCT calcium score shows a linear correlation with echocardiographic and cardiac catheterization parameters [7, 8] indicative of severe AS and with surgical visual assessment of the valve [9]. On these bases, CCT should be able to overcome the diagnostic limitation in the setting of LFLG AS without FR and to give a static, morphological severity score. Calcium score cut-off values for severe AS have been validated on normal flow-normal gradient AS according to the different degrees of AS. Two different studies [8, 10] successively confirmed the need for different gender cuf-off values (men > 2000 A.U., women > 1200 A.U.). A total of 794 patients with different degree of AS at echocardiography underwent a CCT and were followed up for all-cause death in a large observational study [11]. Severe AV calcification (defined by 1180 A.U. in women and 2050 A.U. in men) was an independent and significant predictor of mortality (*p* 0.001), confirming the diagnostic accuracy of CCT.

### Other imaging techniques

Transesophageal echocardiography (TOE) is a well-known and valid alternative to TTE to assess AV planimetry and provides a good visualization of LVOT. It has even been suggested that TOE could guarantee not only a more accurate planimetric valve area assessment than TTE but also a more reliable LVOT area calculation; consequently, a hybrid approach with TTE-derived VTI and TOE-derived LVOT area would give a more precise estimation of true AVA by continuity equation [12]. Also, the invasive measurements of left ventricle and aortic pressures can give a functional AVA estimation by the Gorlin equation or by the Hakki’s simplified version of the formula, during left heart catheterization. Finally, planimetric and continuity equation-derived AVA obtained by cardiac magnetic resonance (CMR) showed good correlation with TTE and TOE [13]. All these quantification techniques can be useful in the case of poor acoustic window or inadequate Doppler alignment, but none of them is able to give a comprehensive assessment of AS severity grade in the low-flow setting.

How previously observed, a LFLG AS, especially without FR at DSE, gives rise to questions about the amount of LVEF impairment that could be attributed to the AS itself or to other causes. From this point of view, the myocardial tissue characterization provided by CMR can add useful information to understand the nature of LV dysfunction; AS is often associated to late gadolinium enhancement (LGE), usually with a midwall scar, differently from the typical pattern of myocardial infarction. Moreover, the presence of midwall LGE in AS has been associated to a significantly higher rate of 30-day mortality [13]. Although the current guidelines for heart valve disease management [1, 2] do not recognize a role to CMR in the diagnostic workup of AS, this technique might provide adjunctive data to predict the possible benefits of AVR.

## Choosing between invasive and conservative treatment

The diagnosis of severe AS according to the previously described multimodality criteria (irrespective to flow status and LVEF) is essential to take AVR into consideration. The replacement of a non-severely stenotic aortic valve can be evaluated only with another concomitant indication for cardiac surgery [1, 2]. Nevertheless, in the context of reduced LVEF, even those patients with moderate AS could expect a significant prognostic improvement with AVR vs OMT [14, 15]. At this regard, the TAVR-UNLOAD [16] is an ongoing clinical trial that is expecting to clarify the need for an eventual redefinition of the criteria to establish the need for AVR in the setting of impaired LVEF. To date, the diagnosis of true severe LFLG-AS is currently mandatory to discuss an eventual AVR and pseudo-severe LFLG AS should be treated with conventional heart failure medications.

Once the severity of LFLG AS has been confirmed, the benefit/risk ratio of the patient must be taken into account to justify an intervention. First of all, all the available studies agreed on a poor long-term survival when LFLG AS is treated with OMT alone [17–21] (Table 1). On the other hand, the coexistence of severe AS and impaired LVEF determines a not-negligible perioperative mortality risk [18, 19, 21–24] (Table 2). SAVR is still associated with a high short-term risk of death in LFLG AS, even though recent advancements in both surgical and anesthesiologic techniques determined a significant improvement. A large observational study [23] which enrolled 217 cases of LFLG AS between 1990 and 2005 showed an overall mortality of 16% during the first 30 postoperative days, however with a notable reduction in the last decades (20% in the 1990–1999 period, mean Euroscore 8.9% vs 10% since 2000, mean Euroscore 9.2%). According to this study, a NYHA class III or IV, a high EUROSCORE (perioperative mortality of 25% with an EUROSCORE > 10%), an MPG < 20 mmHg, a concomitant multivessel CAD, and the absence of FR at DSE were the major predictors of mortality (*p* < 0.01). The coexistence of an unquestionable prognostic improvement after AVR and a high risk of death linked to an absent FR at DSE finds a match in the current guidelines [1, 2], where there is a strong recommendation for AVR in LFLG AS with FR (class of recommendation I, level of evidence C and I,B, respectively) and a more cautious approach in case of no FR (IIa,C according to ESC, while AHA guidelines do not provide a clear indication). Nevertheless, the mentioned studies considered only SAVR but a lower 30-day mortality rate could be expected with the less invasive TAVI. Among the major RCTs that compared TAVI vs SAVR, only the PARTNER 1 made a sub-analysis on severe LFLG AS patients showing only a small increase of risk with SAVR in comparison with TAVI [20]. This result can be partially dampened by the exclusion of LFLG-AS without FR from the study population. Magner et al. [24] enrolled 225 patients diagnosed with LFLG AS and impaired LVEF, with or without FR; all the patients underwent TAVI and 30-day mortality was 8.9%, slightly higher than those calculated by preoperative surgical risk score (mean STS 7.6%). Of note, the enrolment period was 2006–2014, including the very early stage of TAVI use so the not negligible perioperative mortality rate could be now partially mitigated by progressive increase of expertise and technical improvements in the field of structural interventional cardiology. At this regard, Ribeiro and colleagues [22] enrolled, from 2013 to 2017, a cohort of patients who underwent TAVI for LFLG AS and impaired LVEF, having high average preoperative surgical risk (mean STS score 7.7%, mean EUROSCORE II 10.5%). The 30-day mortality rate was 3.8%, notably lower than the preoperative risk estimation. In any case, TAVI in LFLG AS with impaired LVEF seems to be associated to a significant higher mortality in comparison with classical AS: in a recent large matched cohort observational study [25], 1-year mortality after percutaneous AVR in LFLG AS with EF < 50% was more than double than in high gradient severe AS.

**Table 1:** LFLG AS with reduced LVEF (EF < 50%)—comparison between AVR and OMT

	Population of interest (LFLG AS with reduced LVEF)	Type of AVR	AVR vs OMT HR for death at long-term follow-up
Sato et al. [17]^a^	86	TAVI and SAVR	HR 0.32 [6, 6] *p* < 0,001
Tribouilloy et al. [18]	81	SAVR	HR 0.16 to 5.21 varying with time [0.12–3.16 to 0.21–8.50], *p* < 0.00026^b^
Monin et al. [19]	136 pt	SAVR	HR 0.3 (*p* 0,001)
Herrmann et al. [20]	42 pt (considering only inoperable true severe LFLG AS with low EF, cohort B)	TAVI	HR 0.43; 95% CI [0.19–0.98] P = 0.04
Clavel et al. [21]	101 pt	TAVI and SAVR	HR 0.57 [ 0.40 to 0.82] P = 0.02

^a^Only considering true LFLG AS

^b^Hazard ratio has been obtained with a single-variable Gray model instead of classical Cox model. This statistical approach allows the regression coefficients to change over different time intervals, taking into account the drop of HR for death after perioperative period

**Table 2:** 30 days any cause mortality after AVR in LFLG AS with impaired LVEF (EF < 50%)

	Population of interest (LFLG AS with reduced LVEF)	Type of AVR	Surgical risk estimation	30-day mortality	30-day mortality risk factors
Ribeiro et al. [22]	287	TAVI	STS 7.7%	3.8%	COPD *p* 0.022 Anemia *p* 0.004
Tribouilloy et al. [18]	81	SAVR	-	22%	Associated CABG (*p* 0.007) -MPG < 20 mmHg (*p* 0.035)
Monin et al. [19]	136	SAVR	-	14%	-Lack of FR (*p* 0.001) -MPG < 20 mmHg (*p* 0.04)
Clavel et al. [21]	101	TAVI and SAVR	-	18%	-
Levy et al. [23]	217	SAVR	Euroscore 8.9%	16%	-
Magner et al. [24]	225	TAVI	STS 7.6%	8.9%	-

Moreover, the prognostic role of FR at DSE has been recently questioned by several works [5, 13, 26, 27] and new proposed echocardiographic flow rate-based tools seem to provide a better risk estimation than the classic SVi increasement during dobutamine infusion. In the previous mentioned TOPAS-TAVI study [22], the patients underwent DSE (44% LFLG AS with FR, 56% LFLG AS without FR) before TAVI and FR were actually not correlated to the 2-year cardiovascular mortality (*p* = 0.38). Sato et al. [17] followed up 235 patients with LFLG AS and reduced LVEF for a median follow-up of 2.3 years (including, according to DSE results, pseudo-severe AS, true severe AS, and indeterminate AS). AVR (SAVR and TAVI) was performed in 128 patients and the study failed to demonstrate any significant survival difference between patient with or without FR treated with OMT vs AVR; on the contrary, an AVAproj < 1 cm^2^ well predicted the outcome: the patients with an AVAproj < 1 cm^2^ treated with AVR had a significant higher survival than those medically treated. Similarly, in a cohort of 218 patients with resting low gradient AS (resting MPG < 40 mmHg and AVA < 1 cm^2^) undergoing AVR, a low FLR at rest was an independent predictor of the medium-term mortality [27], while absent FR was not. These results can possibly be explained by a superiority of these new parameters in the identification of patients with a true severe AS, so having a real expected benefit from AVR. The reliability of FR in demonstrating a myocardial damage and, consequently, a benefit from AVR, has also been challenged by CMR. Rosa et al. [28] enrolled 41 LFLG AS and 24 normal flow-high gradient AS, all undergoing CMR; the LFLG group underwent DSE to assess the FR, too. The LFLG AS group had more myocardial fibrosis (expressed by extracellular volume and LGE) than the control group, but among LFLG AS, a significant difference between patients with and without FR did not emerge.

**Fig. 1:**
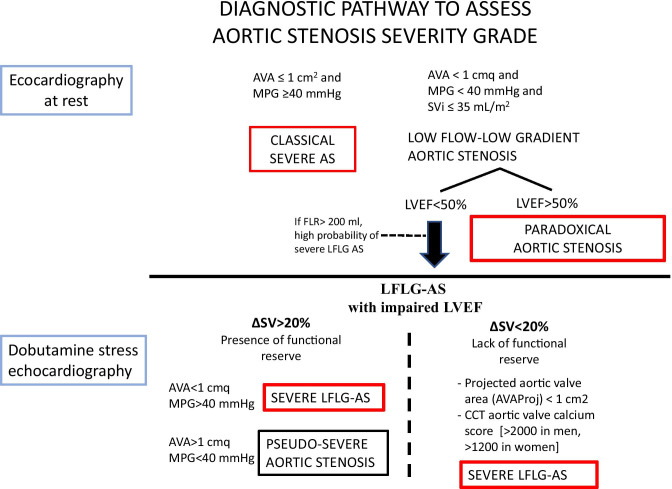
Proposed diagnostic workup to assess the severity grade of low-flow low-gradient aortic stenosis, including the emerging flow rate-based echocardiographic tools (AVAproj and resting FLR). Abbreviations: AVA: aortic valve area; AVAproj: projected aortic valve area; CCT: cardiac computed tomography; FLR: transaortic flow rate at rest; MPG: trans-aortic mean pressure gradient; SVi: stroke volume index; ΔSV: stroke volume variation between rest and stress

Finally, although large trials comparing OMT, TAVI, and SAVR in LFLG AS are not available yet, OMT alone seems to be reserved to patients at very high perioperative risk and short life expectancy. At the same time, it is unquestionable that AVR in LFLG AS with reduced LVEF is affected by worst short- and long-term outcomes in respect to severe AS with preserved EF, irrespectively, to the adopted treatment strategy. Nevertheless, the absence of FR at DSE should not be considered the best predictor of a poor response to AVR, anymore. An accurate selection of patients should include all the other mentioned parameters (CCT calcium score, AVAproj, FLR) in addition to the standard ones with the aim to demonstrate a truly severe AS which will prognostically benefit from AVR (Fig. 1 ).

## Choosing between surgical and transcatheter AVR

**Fig. 2:**
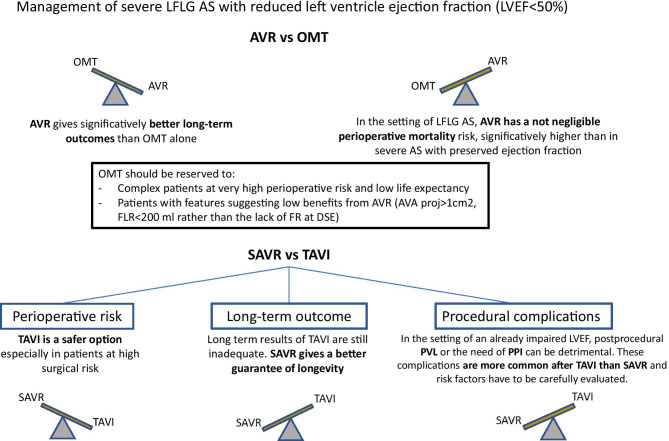
Proposed algorithm for the management of severe low-flow low-gradient aortic stenosis. Abbreviations: AVR aortic valve replacement, AVAproj: projected aortic valve area; DSE: dobutamine stress echocardiography; FLR: transaortic flow rate at rest; FR: functional reserve; OMT: optimal medical therapy; PPI: permanent pacemaker implantation; PVL: paravalvular leak; SAVR: surgical aortic valve repair; TAVI: transcatheter aortic valve repair

To date, there is not a defined algorithm to guide the choice between SAVR and TAVI, and the Heart Team specialists have to make each single case-tailored evaluation. When both the therapeutic options are available (anatomical feasibility criteria for TAVI go beyond the purpose of this paper), the main questions to face include long-term outcomes and assessment of the risk factors for procedure-related complications. Specifically, in the setting of an already impaired LVEF, the risk of paravalvular leak (PVL) and conduction disturbances after TAVI deployment have to be carefully evaluated, because of their nonnegligible prognostic implications (Fig. 2).

### Perioperative risk vs long-term outcomes

How previously observed, despite solid statistical evidences of perioperative risk reduction are still missing, TAVI is an attractive therapeutic option for LFLG AS, especially for patients at high surgical risk. Whether there could be a significant difference about long-term outcome is still an open question. Historically, the approval process for TAVI clinical use went through RCTs comparing the outcomes of patients with different surgical risk treated with TAVI vs SAVR. According to these studies [29–35], TAVI is not inferior for 1-year survival rate, regardless of surgical risk. Moreover, in high-risk and intermediate-to-high-risk patients, the transcatheter approach demonstrated a non-inferiority to SAVR at 2 and 5 years, too. The mentioned trials had a non-inferiority statistical design for a composite cardiovascular outcome (except for SURTAVI [32] which failed to show superiority of TAVI anyway). Nevertheless, a recent meta-analysis [36] including the 7 major RCTs comparing TAVI and SAVR outcomes concluded that TAVI, via transfemoral approach (known to be the safest one), was associated with the higher survival benefit on a 2-year follow-up (17% of death risk reduction [HR 0.83 (95% CI 0.72–0.94)), regardless of surgical risk. It should be observed that although the RCTs with the longer follow-up (5 years) showed a non-inferiority to SAVR, the survival benefit of the percutaneous approach vs surgery tended to decrease over time [29–35, 37–39] (Table 3). A small window on the behavior of TAVI after 5 years from implantation was provided by the UK TAVI register: among 241 patients, only 22 (9.1%) had a moderate-to-severe structural valve degeneration [40]; nevertheless, comparing this data with the one regarding surgical prostheses is difficult mostly because of the inhomogeneous criteria of valve degeneration adopted by literature. The concerns about TAVI longevity in low-risk patients with good life expectancy could be mitigated by the possibility to treat bioprosthetic degeneration with another transcatheter valve implantation. To date, *TAVI in TAVI* procedures are anecdotical, but the extension of the indication to intermediate- and low-risk patients will probably increase the experience in the near future. Nevertheless, we can learn from the percutaneous treatment of degenerated surgically implanted bioprostheses (*TAVI valve-in-valve procedure*), already extensively described. The pivotal limiting factor for a second TAVI is the risk of coronary obstruction due to the displacement of the leaflets of the first bioprosthesis: a short and narrow aortic root represents an important risk factor [41]. Interestingly, different device combination could play a role in the success of *TAVI in TAVI* procedure and, in the future, might influence the choice of the first device to implant [42].

**Table 3:**
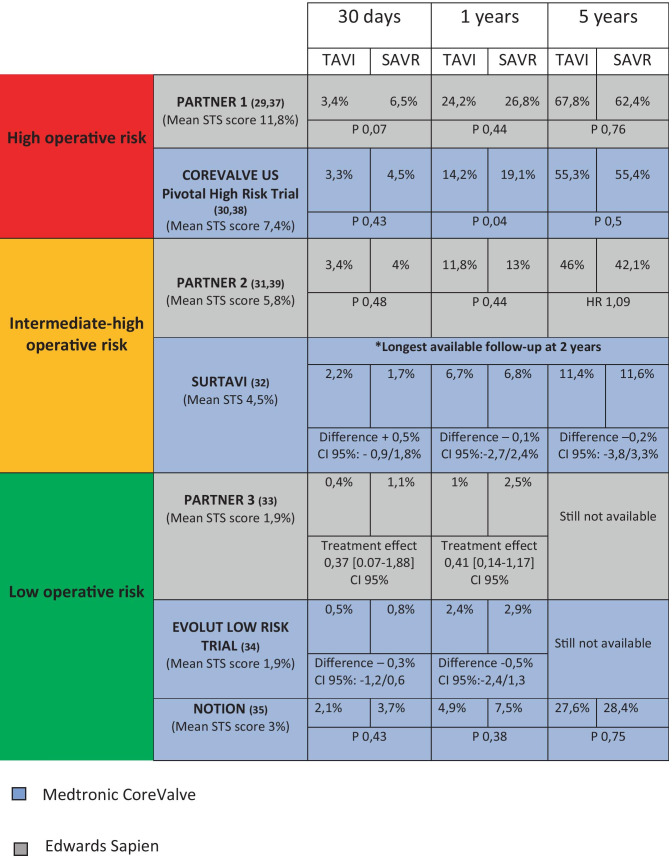
TAVI vs SAVR overall mortality rate according to the majors TAVI RCTs

Even if TAVI is now an available option regardless of the surgical risk score, its use has to be cautious, especially in young and low-risk patients.

### Paravalvular leak

PVL after TAVI is a common procedure-related complication: the overall incidence ranges between 50 and 85%, significantly higher than PVL after SAVR (attested between 1 and 47.6%), even though the majority of TAVI-related PVL is mild (7.8–40.8%) [43]. More than mild PVL has been associated with increased mortality in the overall TAVI population and a residual transaortic regurgitation can be especially detrimental for LFLG AS with an already dysfunctional heart [44]. Therefore, we have to take into account specific anatomical factors related to PVL while deciding between TAVI or SAVR. Shape, diameter, and extensive valve calcification of the aortic annulus are recognized risk factors: transcatheter bioprostheses can adapt with difficulty their rounded shape and anchor to an oval and wide aortic annulus, especially in presence of a high burden of calcium [45]. Even the type of TAVI device can play a role: the CHOICE trial [46] compared second generation balloon expandable vs self-expandable prostheses (Edwards Sapien XT and Medtronic CoreValve) showing a higher incidence of 30-day PVL in the self-expandable group (incidence of more-than-mild PVL at angiographic assessment 4.1% for Edwards vs 18.3% for Corevalve, *P* < 0.001). Notably, the 71% of more-than-mild Corevalve-related PVL downgraded to mild at 1 year: this data related with the post-deployment radial force that the self-expandable device exerts on the aortic annulus. Nevertheless, the CHOICE trial described a higher progression of mild PVL to more severe degrees in the Corevalve group, defining a higher overall risk of PVL with CoreValve than with Edwards bioprostheses (1.1% vs. 12.1%; *P* = 0.005) [47]. Third generation TAVI (Edwards S3 and CoreValve Evolut R/PRO) has been specifically designed to reduce the incidence of aortic regurgitation and a recent study [48] confirmed better outcomes with both bioprostheses. CoreValve showed again a higher incidence of regurgitation but without a significant difference with the Edwards prostheses (incidence of more-than-mild PVL was 8.2% in CoreValve group vs 4.1% in Medtronic group, *p* < 0.1) [48].

Bicuspid aortic valve (BAV) shows all of the three mentioned risk factors for PVL (elliptical annulus shape, wide annulus, and massive and asymmetric calcifications) and is also associated to prosthesis under-expansion, correlated to the risk of an accelerated valve deterioration. Even if according to actual guidelines [1, 2], SAVR is the treatment of choice for BAV, the experience with percutaneous treatment is increasing, mostly consequently to several recent studies that failed to show significant outcome differences in respect to the replacement of tricuspid aortic valve (TAV). The largest available study on 5412 patients [49] comparing TAVI in BAV vs TAV showed a slightly higher incidence of residual moderate or severe PVL in BAV (2.7% vs 2.1%; *P* < 0.001) but with a significant incidence reduction with new generation devices (Sapien 3 and Evolut R, 2.7% vs 14%; *P* < 0.001). Another smaller (but with a propensity score-matched cohort analysis) observational study [50] showed similar results: an overall more frequent adverse procedural events in BAV group, without significant differences between BAV and TAV when new devices were used, irrespectively of the type of implanted prosthesis (Edwards Sapien 3, CoreValve Evolut R or Boston Scientific Lotus). Today, TAVI in BAV is a valuable option, especially when recurring to new generation prostheses. Nevertheless, the procedure deserves a carefully preoperative evaluation. In particular, the assessment of prosthesis size can be challenging even with preoperative CCT because of the distortion of BAV annulus; in this case, an intraoperative balloon sizing can avoid an oversized prosthesis. There are still doubts about the impact of BAV morphology on the procedural success: the presence of a calcified raphe has been linked to a higher incidence of PVL and permanent pacemaker implantation (PPI) [51]. Finally, it should be noticed that the patients with BAV who require AVR are medially younger than TAV patients; therefore, the considerations about life expectancy and prosthesis longevity have to be selectively made.

### Conduction disturbances

PPI after AVR has been recently linked to a reduced long-term survival [52, 53]. The risk of post-procedural need for PPI acquires a particular role during the preprocedural evaluation of LFLG AS. In fact, on the basis of the BLOCK-HF RCT results, latest pacing guidelines [54] suggest to prefer cardiac resynchronization therapy (CRT) to right ventricle pacing alone in patients with an impaired LVEF (EF < 50%). PPI need is relatively uncommon after SAVR (2–5%); on the contrary, high-degree atrioventricular conduction disturbances are habitual complications after TAVI. According to the largest available meta-analyses [55, 56], the estimation of the real incidence of post-TAVI PPI is difficult because of the large inhomogeneity of the results of the studies and the evolution of the devices. The major risk factors are: pre-existing conduction disturbances (especially first-degree atrio-ventricular block, left anterior fascicular block, and right bundle branch block), extensive aortic annulus calcifications (especially at the non-coronary cusp landing zone), pre-deployment valvuloplasty, deep device implantation, short length of membranous septum, and implantation of self-expandable devices [54, 55, 57]. Traditionally, balloon expandable prostheses have been associated with a lower rate of conduction disorders than the self-expandable ones but this gap is reducing with the new generation devices [54]. Despite PPI is not a negligible risk when TAVI is performed, the real need of long-term pacing has been recently questioned [58]: patients who receive pacemakers for intermittent conduction disturbances are most of the time no-pacing dependent at the following controls, suggesting the possible prolongation of the observational period before definitive electrical therapy.

New onset bundle branch blocks (especially left bundle branch block, LBBB) can be particularly detrimental in LFLG AS too; in fact, LBBB-related electrical and mechanical dyssynchrony can severely reduce the benefit of the afterload reduction obtained with AVR. New onset of LBBB after SAVR ranges between 4.4 and 4.6% [59, 60]. Nevertheless, new sutureless and rapid deployment surgical bioprostheses can markedly increase conduction disturbances, reaching an incidence of 16% [60]. Incidence of post-TAVI LBBB onset varies among papers, ranging from 15 to 20% [61, 62] with the same risk factors as PPI. There is still not consensus about the impact of TAVI-related branch blocks on patient prognosis. A large observational study which followed up 1020 patients treated with TAVI for a median time of 3 years failed to demonstrate a significant difference in terms of cardiovascular mortality or HF rehospitalization between patients with and without post-procedural LBBB [61], even if the LBBB group showed lower LVEF recovery. On the other hand, a sub-analysis from PARTNER II trial [62] followed up 1179 intermediate surgical risk patients treated with TAVI for 2 years showing significantly higher cardiovascular mortality and HF rehospitalization in new onset LBBB group. Although the baseline characteristics of the two study populations were similar, the overall mortality was particularly higher in the first study (43% vs 11.7%) and this could have influenced the prognostic impact of procedure-related LBBB. To date, there are few only anecdotical reports available about CRT after TAVI-related LBBB with doubts related to the right timing and the real utility of CRT in this specific population. Usually, the recovery of LVEF occurs in the very first days after the procedure and the long-term follow-up does not show significant changes [18, 63]. Therefore, early CRT implantation seems a reasonable option when early post-procedural echocardiography does not demonstrate a satisfactory LV function recovery. However, a recent study [64] questioned the usefulness of electrical resynchronization of AVR-related conduction disturbances: 140 patients were followed up for 19 ± 9 months after TAVI, among which the 20% developed LBBB; 26 (98%) did not meet echocardiographic criteria to define a dyssynchronous LBBB contraction pattern and there was no significant overall mortality difference between the two groups.

### Conclusion

The management of LFLG AS with impaired LVEF is still challenging. Cardiologists have several tools to quantify the contribution of AV disease to the LV dysfunction and the lack of contractility reserve per se should not discourage further investigations to evaluate the possible benefit of the valve replacement. True severe LFLG AS has very poor prognosis on OMT alone and abstention from intervention should be reserved only to extremely critical patients. The coexistence of severe AS and impaired LVEF always concerns the Heart Team specialists and pushes towards a transcatheter approach that currently seems to guarantee a lower perioperative risk than the surgical treatment. To date, there are not enough data available about long-term outcome of TAVI. Therefore, especially in young subjects who are expected to gain a significant benefit from AVR and with a good life expectancy, the choice of the best treatment has to be carefully evaluated, even discussing with the patient itself about pros and cons of both solutions. Finally, the decision between TAVI and SAVR imposes a comprehensive short-term and long-term risk evaluation, especially when pre-existent factors can predispose to a suboptimal transcatheter treatment. Further studies and sub-analysis of the large available RCTs are needed to help clinicians in the management of this LFLG AS with reduced LVEF.
